# Hematological Prognostic Scoring System Can Predict Overall Survival and Can Indicate Response to Immunotherapy in Patients With Osteosarcoma

**DOI:** 10.3389/fimmu.2022.879560

**Published:** 2022-05-06

**Authors:** Longqing Li, Yang Wang, Xuanhong He, Zhuangzhuang Li, Minxun Lu, Taojun Gong, Qing Chang, Jingqi Lin, Chuang Liu, Yi Luo, Li Min, Yong Zhou, Chongqi Tu

**Affiliations:** ^1^ Department of Orthopedics, Orthopaedic Research Institute, West China Hospital, Sichuan University, Chengdu, China; ^2^ Bone and Joint 3D-Printing and Biomechanical Laboratory, Department of Orthopedics, West China Hospital, Sichuan University, Chengdu, China; ^3^ Institute of Jinan Yinfeng Medical Laboratory, Yinfeng Gene Technology Co Ltd, Jinan, China

**Keywords:** osteosarcoma, hematological, prognostic, inflammation, immunotherapy

## Abstract

Osteosarcoma is the most common primary malignant bone tumor with a high metastatic potential. Nowadays, there is a lack of new markers to identify prognosis of osteosarcoma patients with response to medical treatment. Recent studies have shown that hematological markers can reflect to some extent the microenvironment of an individual with the potential to predict patient prognosis. However, most of the previous studies have studied the prognostic value of a single hematological index, and it is difficult to comprehensively reflect the tumor microenvironment of patients. Here, we comprehensively collected 16 hematological markers and constructed a hematological prognostic scoring system (HPSS) using LASSO cox regression analysis. HPSS contains many indicators such as immunity, inflammation, coagulation and nutrition. Our results suggest that HPSS is an independent prognostic factor for overall survival in osteosarcoma patients and is an optimal addition to clinical characteristics and well suited to further identify high-risk patients from clinically low-risk patients. HPSS-based nomograms have good predictive ability. Finally, HPSS also has some hints for immunotherapy response in osteosarcoma patients.

## Introduction

Osteosarcoma is a rapidly progressive primary malignant bone tumor with high metastatic potential, accounting for 20% to 40% of all bone tumors (1, 2). Chemotherapy treatment, introduced in the 1970s, significantly improved the five-year survival rate of patients with non-metastatic osteosarcoma (3). However, approximately 15 – 20% of affected patients already have metastases at presentation and individuals with metastatic disease have low short- and long-term survival (4–6). In addition, tumor recurrence and chemoresistance are also recognized as important prognostic factors (7, 8). These clinical features are important in distinguishing high-risk patients and guiding treatment (9). However, the progression of the disease may be distinct in patients with similar clinical features. Therefore, more factors need to be considered to facilitate precision treatment.

Immunotherapy has shown definite clinical benefit in some advanced solid tumors (10, 11). Osteosarcoma has relatively high programmed cell death 1 ligand-1 (PD-L1) expression and may therefore benefit from immunotherapy (12, 13). Unfortunately, several recent clinical trials have shown that immunotherapy does not achieve the desired efficacy in osteosarcoma (14, 15). Therefore, effective biomarkers may be needed to identify patients who may truly benefit from this therapy (16).

Recent studies have shown that preoperative hematological markers such as neutrophil to lymphocyte ratio (NLR), platelet to lymphocyte ratio (PLR), and lymphocyte to macrophage ratio (LMR) can reflect the individual’s tumor microenvironment to some extent and can be used to predict the prognosis of cancer patients (17, 18). These hematological markers are readily available and cost-effective and are ideal prognostic markers. Many recent studies have confirmed the value of these markers in predicting survival and response to medical treatment in cancer patients, including osteosarcoma (19–21).However, single hematological markers have shortcomings such as insufficient prognostic power and instability. Therefore, overcoming these shortcomings will help to improve the value of hematological markers to promote their utilization.

In this study, we collected proven prognostic hematological marks and developed hematological prognostic scoring system (HPSS) by iterative least absolute contraction and selection operator (LASSO) COX proportional hazards regression analysis. Our study shows that HPSS overcomes the disadvantages such as insufficient predictive power and instability of a single hematological marker, and is an effective supplement to clinical features.

## Patients And Methods

### Patients

With the approval of the Medical Ethics Committee, we reviewed the clinical data of osteosarcoma patients from January 2016 to January 2021 in the database of the Musculoskeletal Tumor Center of West China Hospital. During the review process, we included and excluded patients according to the following criteria: 1) patients with high grade osteosarcoma confirmed by histopathology; 2) patients have complete hematological test results before neoadjuvant chemotherapy; 3) patient received standard treatment at West China Hospital. The exclusion criteria:1) Patients with histopathologically confirmed low-grade osteosarcoma (intramedullary and bone surface) and periosteal osteosarcoma; 2) Patients who had received neoadjuvant chemotherapy before their first-time consultancy in our hospital; 3) patients with hematological diseases; 4) patients with other malignancies; 5) patients not received standard treatment (patients who are misdiagnosed and mistreated or fail to complete postoperative chemotherapy). Finally, 223 patients were included in our study after passing the inclusion and exclusion criteria. Each patient was followed up regularly until death or January 2022. The following follow-up principles were followed: reexamination every 3 months within 1 year after surgery; reexamination every 4 months 1-2 years after surgery; reexamination every 5 months 2-3 years after surgery; reexamination every 6 months 3-5 years after surgery; reexamination every year more than 5 years after surgery. All patients were randomly divided into a training set (n=156, 70%) and external validation set (n=67, 30%) using a random seed set in 2022.

In addition, 14 patients with metastatic advanced osteosarcoma treated with PD-L1 agents were included in the study. These patients were all tested for PD-L1 expression at a third-party testing facility (Institute of Ji’nan Yin Feng Medical Laboratory) and had a Tumor Proportion Score > = 1% (TPS) (22). TPS is defined as the proportion of tumor cells positive, i.e., number of tumor cells with positive PD-L1 membrane staining at any intensity/total number of tumor cells * 100%. The TPS of all patients was evaluated by two experienced pathologists. Recombinant Anti-PD-L1 Antibody 28-8(Abcam) was used in all patients; Patients lost operative indicatio and received standard chemotherapy before immunotherapy and had measurable lesions. All patients were administered with camrelizumab (Jiangsu Hengrui Medicine Co., Ltd. China) at a dose of 2 mg/kg every 21 days. All patients underwent a minimum of 2 cycles of immunotherapy. Patients were assessed for efficacy based on RECIST by two researchers not associated with this study.

### Data Collection and Processing

Neutrophils count (Neut#), lymphocytes count (LYMPH#), monocytes count(MONO#), platelet count (PLT), hemoglobin (HB), red blood cell distribution width-coefficient of variation (RDW-CV), red blood cell distribution width-standard deviation (RDW-SD), albumin (A), lactate dehydrogenase (LDH), alkaline phosphatase (ALP), activated partial thromboplastin time (APTT), prothrombin time (PT), thrombin time (TT), fibrinogen (FIB) and international normalized ratio (INR) were extracted from the first blood routine, coagulation function tests and liver and kidney function of 223 patients before neoadjuvant chemotherapy. The formulas for calculating NLR, PLR, LMR, PNI, SII and SIRI are as follows: NLR = Neut#/LYMPH#, PLR = PLT/LYMPH#, LMR = LYMPH#/MONO#, PNI = A + 0.005* LYMPH#, SII = PLT*Neut#/LYMPH#, SIRI = Neut#* MONO#/LYMPH#. In addition, age, gender, tumor location, pathologic fracture status, and tumor metastasis status were abstracted from the patients’ medical records. Overall survival (OS) was calculated from the date of tumor resection to the date of last follow-up or death. In the overall cohort, the optimal cutoff value for each hematological marker was calculated based on the “tdROC” package and converted into a binary variable according to the cutoff value. The 14 patients with osteosarcoma treated with camrelizumab had their hematological markers collected before the start of immunotherapy, and the haematological markers were divided into dichotomous variables following the same cutoff value as the overall cohort.

### Development and Validation of the HPSS

First, univariate cox regression analysis was used to screen indicators of prognostic value in the overall cohort. Based on prognosis-related hematological markers, LASSO cox regression analysis was performed on the training set to determine the optimal hematological prognostic scoring system (HPSS). The LASSO model is an estimation method that enables the reduction of the set of indicators. LASSO regression has the advantages of ridge regression and subset selection at the same time, which makes it superior to other methods in terms of prediction accuracy and model interpretability for high-dimensional multicollinearity problems. The HPSS was calculated for each patient in the training and validation sets based on the coefficients assigned by the LASSO cox regression analysis. Receiver operating curves were used to compare HPSS to the individual hematology markers in both training and validation sets. In the training set, the optimal cutoff value was calculated for HPSS using the survivalROC “package, and the patients were divided into high-risk and low-risk groups based on the cutoff value. The same cutoff was used for the validation set. Kaplan-Meier survival curves were plotted to show the difference in OS between the two groups of patients. Whether HPSS is an independent prognostic factor for predicting OS in osteosarcoma patients was assessed using multivariate cox regression analysis. ROC curves for HPSS versus clinical variables were plotted and contrasted from 1 to 5 years in the training set and the validation set using the timeROC package.

### Construction and Evaluation of the Nomogram

A nomogram was constructed combining HPSS with clinical features in the training set. The discrimination ability and accuracy of nomograms were evaluated by Harrell’s Concordance Index and calibration curve, respectively. Decision curve analysis (DCA) was used to evaluate the clinical application of the nomogram. In addition, the constructed nomogram also predicted the overall survival of the validation cohort to assess the stability of the nomogram’s predictive ability.

### Exploration of the Relationship Between the HPSS and Clinical Characteristics

In all 223 patients, the relationship between the HPSS and traditional clinical features, such as tumor site, pathological fracture, tumor metastasis status, was further researched. At the same time, we divided the patients into four groups by tumor metastasis status, pathological fracture status combined with HPSS, respectively. Two-factor KM survival curves were drawn to show the differences in overall survival among the four groups of patients.

### Assessing Immunotherapy Response Using HPSS

We calculated HPSS for 14 patients with advanced osteosarcoma treated with immunotherapy using the same coefficients as the training cohort. The immunotherapy efficacy of 14 patients was divided into disease control rate (DCR) and progressive disease(PD) based on RESICT. The fisher’s exact test was used to assess the difference between DCR and PD between patients in the high-risk group and those in the low-risk group.

### Statistical Analysis

Kolmogorov-Smirnov was used to assess whether continuous variables were normally distributed, and t-test or Mann-Whitney U test was used to assess differences between continuous variables according to the results. Categorical variables were evaluated using the chi-square test and the fisher’s exact test based on the number of individuals in each group. All statistical analyses were conducted using R software, version 4.1.0 (Institute for Statistics and Mathematics, Vienna, Austria). P values < 0.05 were considered to indicate statistical significance.

## Results

### Patient Characteristics

A total of 223 patients with osteosarcoma were included in the study, including 131 male and 92 female. The age of the patients ranged from 7 to 67 years with a mean age of 21 years. The majority of patients had tumors located in the extremities, and only 9 patients had tumors located in non-extremity sites. A total of 25 patients already had pathological fractures at presentation. In addition, 39 patients had already developed tumor metastasis at presentation. Two hundred twenty-three patients were randomly assigned to the training cohort versus the validation cohort. The demographic and clinical characteristics of the training cohort versus the validation cohort are shown in **Table 1**, with no significant differences between the two groups of cohorts. Optimal cutoff values for 16 hematological markers are provided with **Supplementary Table 1** (ALP, PLR, NLR, SII, FIB, SIRI, PT, HB, APTT, INR, PNI, LMR, RDW-CV, RDW-SD, LDH, TT). As shown in **Table 1**, the distributions of all variables in the training and validation sets are not significantly different.

**Table 1:** Differences in the distribution of all variables between the training set and the validation set and the respective coefficients of the seven hematological indicators that make up the HPSS.

	Train (N = 156)	Test (N = 67)	P-value	Coefficient
**OS.time**				Not applicable
Mean (SD)	1020 (533)	996 (602)	0.787	
**OS**				Not applicable
Alive	101 (64.7%)	46 (68.7%)	0.681	
Died	55 (35.3%)	21 (31.3%)		
**Gender**				Not applicable
Male	89 (57.1%)	42 (62.7%)	0.525	
Female	67 (42.9%)	25 (37.3%)		
**Age**				Not applicable
Mean (SD)	21.8 (12.6)	21.4 (11.7)	0.823	
**Metastasis.status**				Not applicable
No	132 (84.6%)	52 (77.6%)	0.285	
Yes	24 (15.4%)	15 (22.4%)		
**Tumor.site**				Not applicable
Extremities	150 (96.2%)	64 (95.5%)	1	
Non-extremities	6 (3.8%)	3 (4.5%)		
**Pathological.fracture**				Not applicable
No	135 (86.5%)	63 (94.0%)	0.163	
Yes	21 (13.5%)	4 (6.0%)		
**NLR**				Excluded
High	60 (38.5%)	30 (44.8%)	0.464	
Low	96 (61.5%)	37 (55.2%)		
**PLR**				0.521
High	48 (30.8%)	19 (28.4%)	0.841	
Low	108 (69.2%)	48 (71.6%)		
**LMR**				Excluded
High	126 (80.8%)	58 (86.6%)	0.394	
Low	30 (19.2%)	9 (13.4%)		
**PNI**				-0.058
High	98 (62.8%)	44 (65.7%)	0.799	
Low	58 (37.2%)	23 (34.3%)		
**SII**				0.097
High	31 (19.9%)	17 (25.4%)	0.46	
Low	125 (80.1%)	50 (74.6%)		
**SIRI**				Excluded
High	37 (23.7%)	16 (23.9%)	1	
Low	119 (76.3%)	51 (76.1%)		
**HB**				Excluded
High	101 (64.7%)	47 (70.1%)	0.53	
Low	55 (35.3%)	20 (29.9%)		
**RDW-SD**				Excluded
High	58 (37.2%)	16 (23.9%)	0.0753	
Low	98 (62.8%)	51 (76.1%)		
**RDW-CV**				Excluded
High	86 (55.1%)	34 (50.7%)	0.649	
Low	70 (44.9%)	33 (49.3%)		
**PT**				0.051
High	37 (23.7%)	21 (31.3%)	0.306	
Low	119 (76.3%)	46 (68.7%)		
**INR**				Excluded
High	66 (42.3%)	29 (43.3%)	1	
Low	90 (57.7%)	38 (56.7%)		
**APTT**				Excluded
High	62 (39.7%)	23 (34.3%)	0.54	
Low	94 (60.3%)	44 (65.7%)		
**TT**				Excluded
High	84 (53.8%)	35 (52.2%)	0.941	
Low	72 (46.2%)	32 (47.8%)		
**FIB**				0.330
High	135 (86.5%)	58 (86.6%)	1	
Low	21 (13.5%)	9 (13.4%)		
**ALP**				0.785
High	82 (52.6%)	40 (59.7%)	0.404	
Low	74 (47.4%)	27 (40.3%)		
**LDH**				0.186
High	103 (66.0%)	40 (59.7%)	0.453	
Low	53 (34.0%)	27 (40.3%)		

### Establishment and Validation of Hematological Risk Model for Osteosarcoma

**Figure 1:**
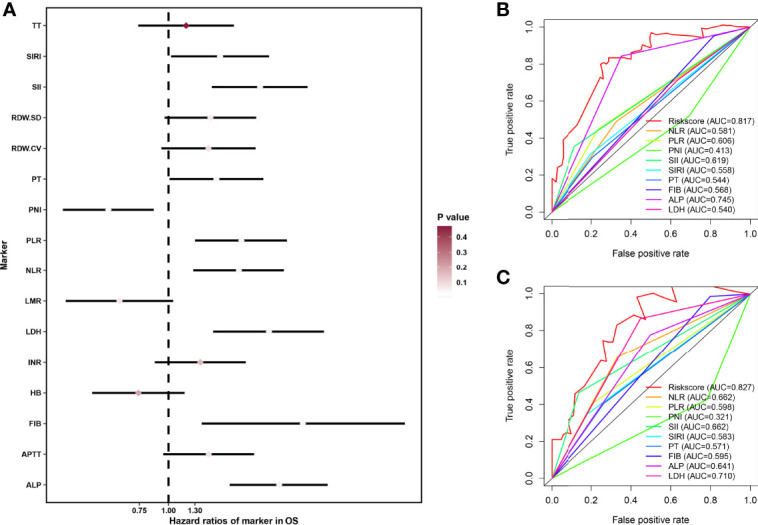
Construction of HPSS and its comparison with individual hematological parameters. **(A)** Forest plot showing the results of univariate cox regression analysis of 16 hematological markers; **(B)** ROC curves showing the predictive power of HPSS in the training set versus a single hematology indicator; **(C)** ROC curves showing the predictive power of HPSS in the validation set versus a single hematology indicator.

First, we performed univariate cox regression analysis of hematologic markers in the overall cohort to determine the association between hematologic markers and OS in patients with osteosarcoma. As shown in **Figure 1**, univariate cox regression analysis showed that 9 hematological markers were statistically significant. As described above, a LASSO cox regression analysis was performed in the training set using 9 hematological indicators and the HPSS consisting of 7 hematological indicators was finally determined. The coefficients for each indicator in the HPSS are shown in **Table 1**, and the HPSS was calculated for each patient based on these coefficients. The results of ROC curves indicated that the predictive ability of HPSS was significantly higher than that of individual hematological markers both in the training and validation cohorts (0.817 vs 0.413-0.745; 0.827 vs 0.321-0.710, **Figures 1B, C**).

**Figure 2:**
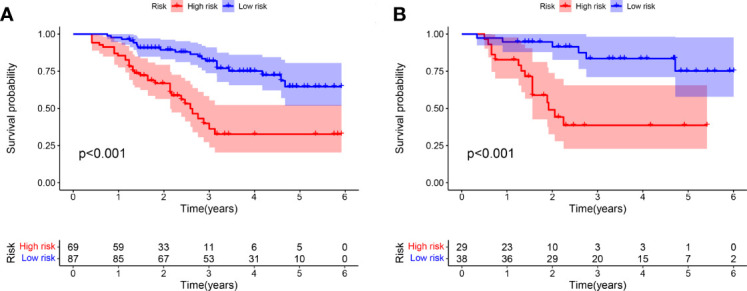
There are significant differences between patients in HPSS risk groups. **(A)** High-risk patients in the training set had significantly lower overall survival than low-risk patients; **(B)** High-risk patients in the validation set had significantly lower overall survival than low-risk patients.

Optimal cutoff values were also calculated for HPSS. The training cohort was divided into two groups with the validation cohort according to the optimal cutoff value. As shown by **Figures 2A, B**, the overall survival of patients in the high HPSS risk group was low in both the training and validation cohorts (P < 0.001).

**Figure 3:**
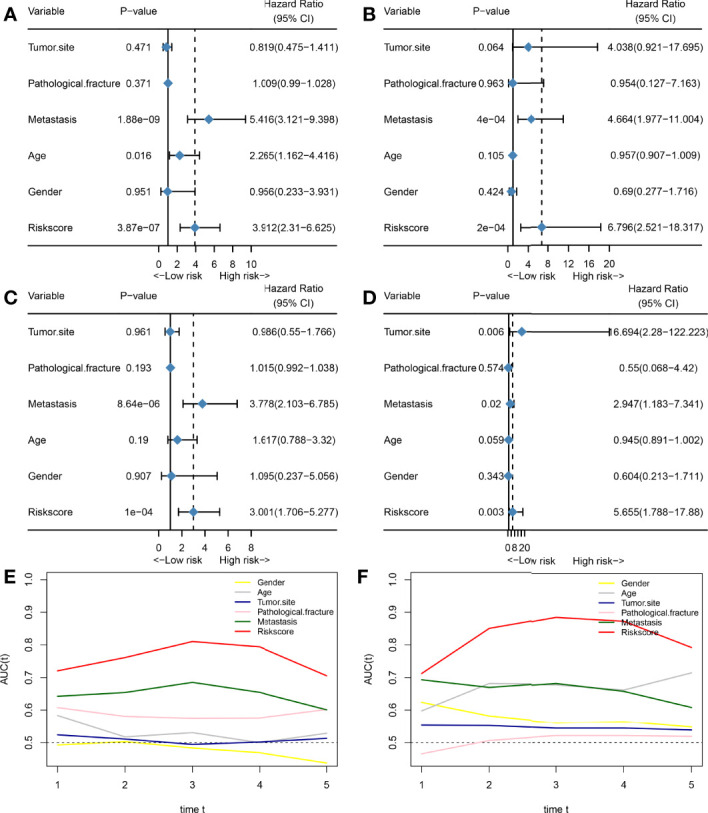
HPSS is an independent prognostic factor for overall survival in patients with osteosarcoma and has certain advantages compared with clinical characteristics. **(A)** Forest plot showing the results of univariate COX regression analysis of HPSS and clinical characteristics in the training set; **(B)** Forest plot showing the results of multivariate COX regression analysis of HPSS and clinical characteristics in the training set; **(C)** Forest plot showing the results of univariate COX regression analysis of HPSS and clinical characteristics in the validation set; **(D)** Forest plot showing the results of multivariate COX regression analysis of HPSS and clinical characteristics in the validation set; **(E)** Time-dependent ROC curves showing the predictive power of HPSS and clinical features in the training set; **(F)** Time-dependent ROC curves showing the predictive power of HPSS and clinical features in the training set; It can be seen that the predictive power of each variable varies over time.

Subsequently, we also assessed whether HPSS was an independent prognostic factor for predicting overall survival in osteosarcoma patients. As shown in **Figures 3A–D**, the results of multivariate cox regression analysis showed that HPSS was an independent prognostic factor for overall survival in osteosarcoma patients in both the training and validation cohorts(training cohort: HR:6.796(2.521-18.317); validation cohort: HR:5.655(1.788-17.88)).

Finally, we plotted time-dependent ROC curves to contrast the predictive ability of HPSS with clinical features such as tumor metastatic status, and pathological fractures. As shown by **Figures 3E, F**, the predictive ability of the HPSS was similar in the change curves of the training and validation cohorts, that is, it was lowest in predicting 1-year mortality, but the predictive ability of the HPSS gradually increased with time. At 2 years and beyond, the predictive power of the HPSS was significantly higher than that of the clinical features.

### Construction and Validation of HPSS-Based Nomograms

**Figure 4:**
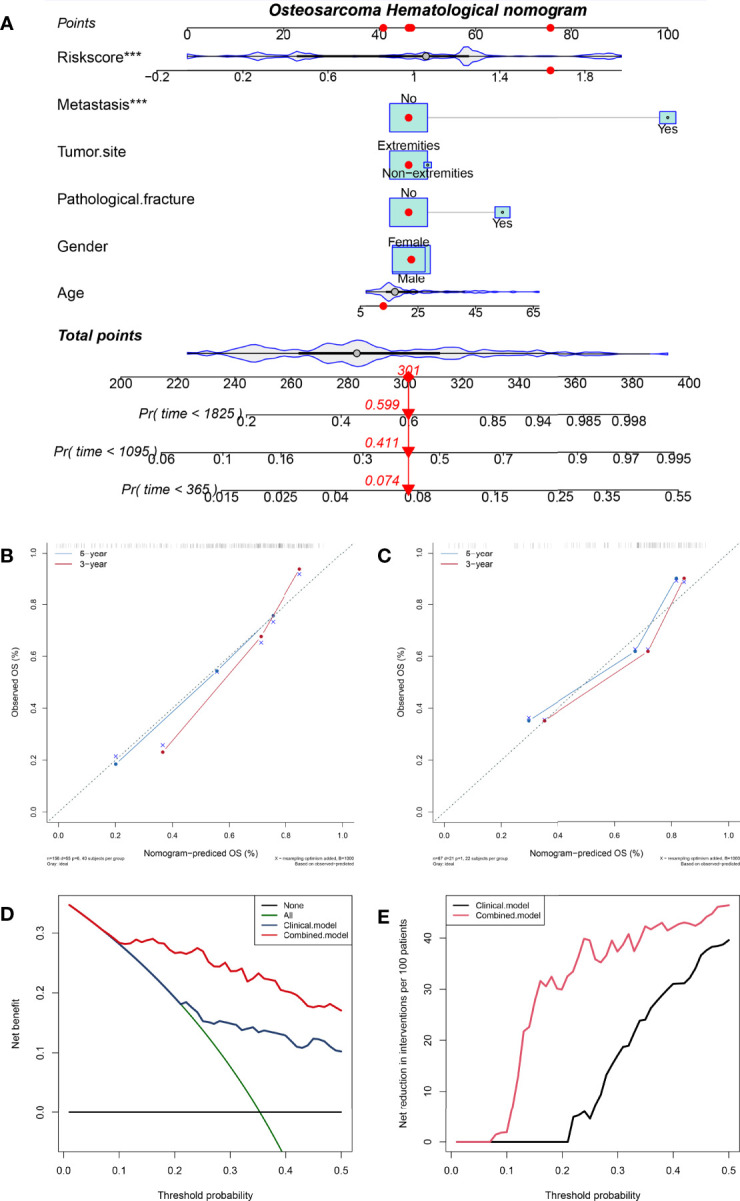
A nomogram was constructed combining HPSS with clinical features and the predictive power of the nomogram was assessed. **(A)** The nomogram of the overall survival of patients with osteosarcoma shows that HPSS score and tumor metastasis status are the two most important variables; **(B)** Calibration curves for nomogram predicting 3-year and 5-year survival of patients in the training set; **(C)** Calibration curves for nomogram predicting 3-year and 5-year survival of patients in the validation set; **(D)** The clinical net benefit curve of the nomogram; **(E)** Clinical Net Reduction Curve for Nomogram. ***p < 0.001

In order to promote the clinical application of HPSS, based on the training cohort, we constructed a nomogram combining HPSS with clinical characteristics. Cox proportional hazards regression assigned a score according to the hazard ratio for each covariate in the nomogram, and the sum of the scores for each covariate was the nomogram total score. The C-index of the constructed nomogram was 0.80, and the calibration curve indicated that the nomogram had good predictive accuracy in predicting 3-year and 5-year overall survival in the training cohort (**Figures 4A, B**). To further validate the stability of the nomogram, we tested the nomogram using the validation cohort. The C-index of the nomogram in the validation set was 0.77, and the calibration curve of the validation set showed that the nomogram still had good predictive ability in the validation cohort (**Figure 4C**). Finally, we explore the clinical benefits of nomograms through clinical decision analysis. Our results suggest that the nomogram added to the HPSS brings significant net benefits over models with only clinical features (**Figures 4D, E**).

### Assessing the Stability of HPSS

**Figure 5:**
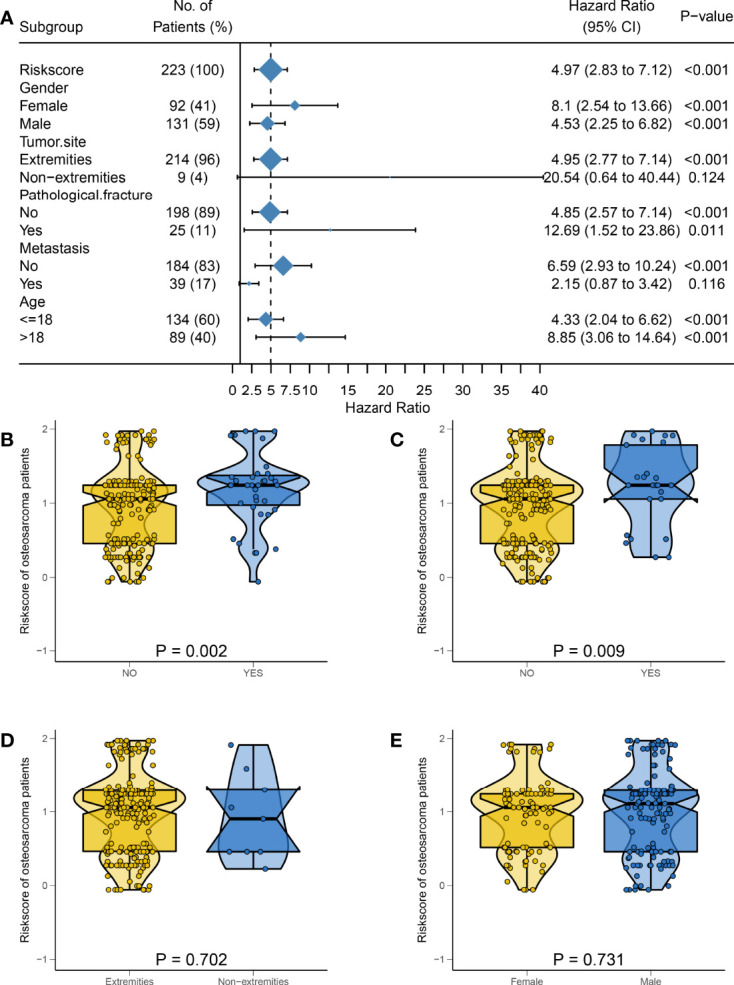
The predictive power of HPSS in subgroups and the relationship between HPSS and clinical characteristics were assessed. **(A)** A forest plot showing the predictive power of HPSS in each subgroup, it can be seen that HPSS has limited predictive power in patients with tumor metastasis and non-extremity groups; **(B)** The relationship between HPSS and tumor metastasis status; **(C)** The relationship between HPSS and pathological fracture status; **(D)** The relationship between HPSS and tumor location; **(E)** The relationship between HPSS and gender.

In order to assess the stability of the HPSS and facilitate its precise application, we set up different subgroups according to clinical characteristics to explore the application of the HPSS in each group. As shown in **Figure 5A**, patients were divided into 10 groups according to age, gender, tumor location, metastatic status and pathological fracture. The predictive ability of HPSS was limited in patients with metastatic group, and non-extremities group. Combined with the previously drawn time-dependent ROC curve, we believe that HPSS should be more used as a supplement to clinical features to further identify high-risk patients from patients in the low-risk group of clinical features.

### Association Between HPSS and Clinical Features

Finally, we further assessed the relationship between HPSS and clinical characteristics. The results of the violin plot indicated that patients in the tumor metastasis group and pathological fracture group had higher HPSS scores (metastasis: P = 0.002; pathological fracture: P= 0.009). However, there was no significant difference in HPSS between patients with different gender, tumor location (**Figures 5B** **–E**).

**Figure 6:**
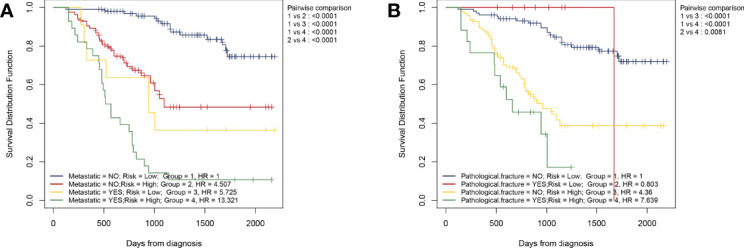
Simple combination of HPSS and clinical features can better predict the prognosis of patients with osteosarcoma. **(A)** Patients with osteosarcoma can be divided into four groups according to tumor metastasis status and HPSS risk, and the KM survival curve shows the difference in survival among the four groups; **(B)** Patients with osteosarcoma can be divided into four groups according to pathological fracture status and HPSS risk, and the KM survival curve shows the difference in survival among the four groups.

As mentioned above, we believe that HPSS is the best complement to clinical features. Therefore, we combined HPSS with tumor metastasis status and divided patients into four groups to assess differences in patient survival. As shown, there was a significant difference in survival among the four groups. Among them, patients in the high HPSS risk group among patients in the non-metastatic group had significantly lower overall survival than those in the low HPSS risk group. Finally, we combined HPSS with pathological fracture status with the same method to obtain similar conclusions (**Figures 6A, B**).

### Assessing Immunotherapy Response Using HPSS

**Figure 7:**
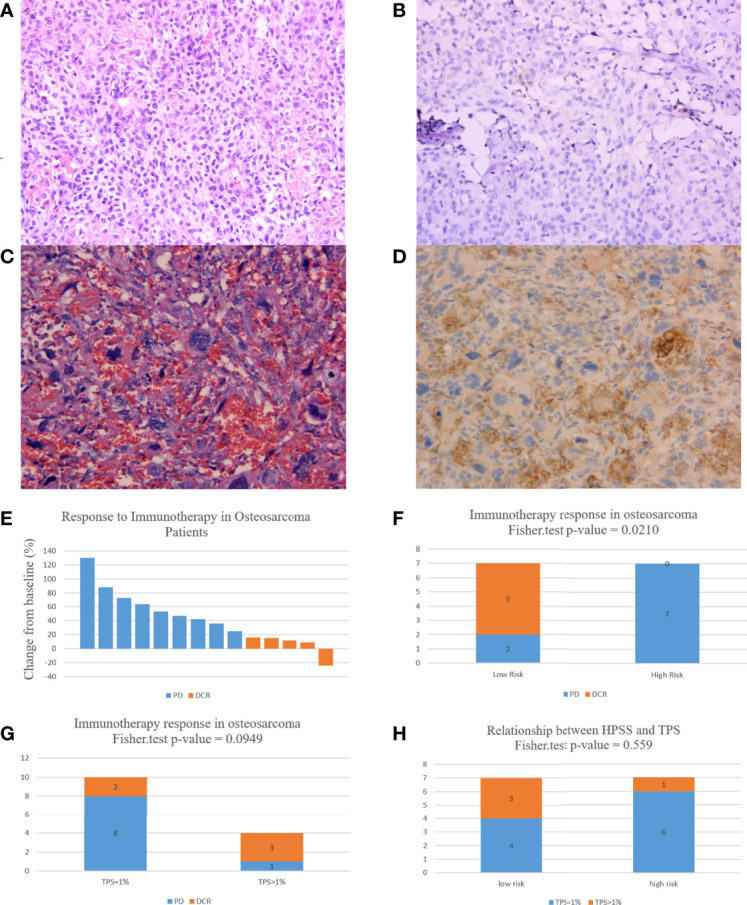
HPSS can predict patient response to immunotherapy to a certain extent. **(A)** HE staining of a patient with 3% TPS expression; **(B)** PD-L1 expression in a patient with a TPS expression of 3; **(C)** HE staining of a patient with 8% TPS expression; **(D)** PD-L1 expression in a patient with a TPS expression of 8; **(E)** A waterfall plot of the response to immunotherapy in 14 osteosarcoma patients; **(F)** Histogram showing differences in immunotherapy status in HPSS risk groups; **(G)** Histogram showing differences in immunotherapy status in different TPS groups; **(H)** Differences in TPS values in different HPSS risk groups.

As described above, all patients had TPS ≥ 1%, however only 4 patients had TPS > 1%; these 4 patients had TPS of 8%, 5%, 3%, and 2%, respectively, and the remaining patients had TPS = 1%. **Figures 7A**–**D** presents the PD-L1 immunohistochemistry results of the 2 patients. According to RECIST, 9 of 14 patients developed PD and only 5 patients were assessed as DCR (**Figure 7E**). Based on HPSS, 7 patients were high risk and 7 patients were low risk. PD occurred in all patients in the high-risk group, and only 2 in the low-risk group. The results of Fisher’s exact test suggest that HPSS can predict the response to immunotherapy to a certain extent (p = 0.0210, **Figure 7F**).

**Figure 8:**
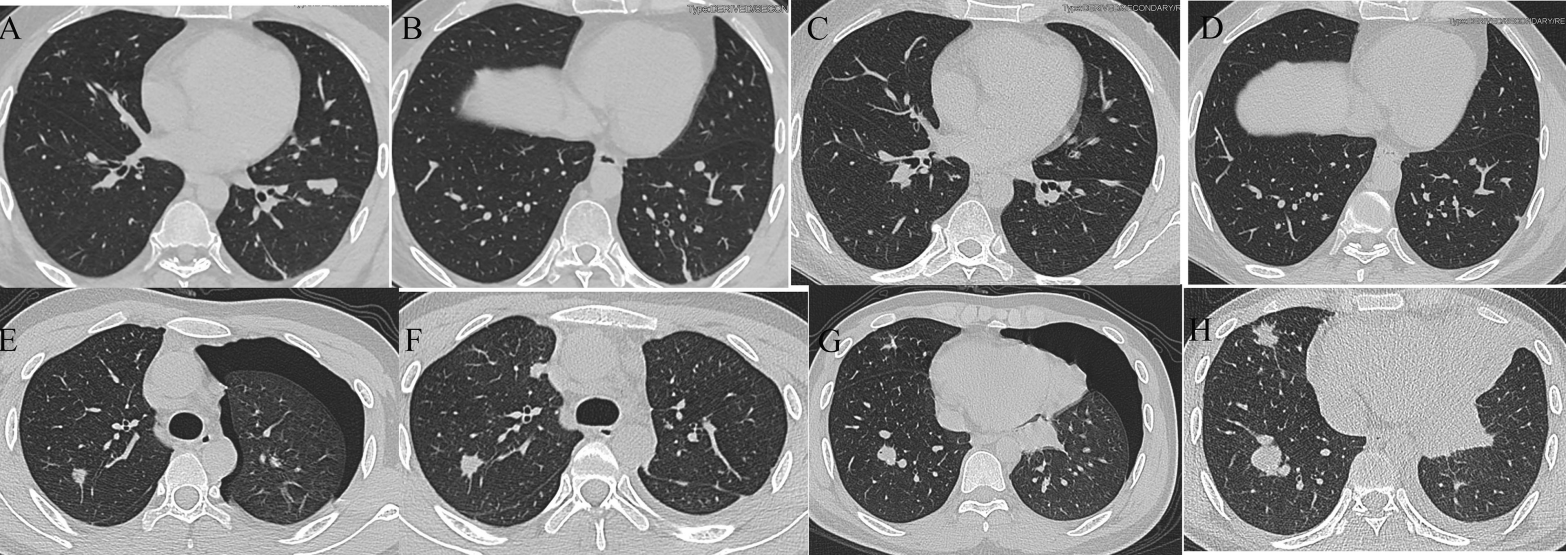
Lung CT results of one PD and one DCR patient. **(A, B)** Lung CT results of a DCR patient before immunotherapy; **(C, D)** Lung CT results of a DCR patient after immunotherapy; **(E, F)** Lung CT results of a PD patient before immunotherapy; **(G, H)** Lung CT results of a PD patient after immunotherapy.

In addition, we evaluated the relationship between TPS and response to immunotherapy. DCR was achieved in 3 of 4 patients with TPS > 1% and in only 2 of 10 patients with TPS = 1%. Unfortunately, this difference did not reach statistical significance (p = 0.0949, **Figure 7G**). Finally, we also assessed the relationship between HPSS and TPS. Our results indicated no significant relationship between HPSS and TPS (p = 0.559, **Figure 7H**). **Figure 8** shows lung CT results before and after drug treatment in a PD patient and a DCR patient.

## Discussion

With the continuous development of surgical techniques and the continuous emergence of various novel treatments, the mortality rate of cancer patients is gradually decreasing. However, since the 1970s, the overall survival of osteosarcoma patients has reached a bottleneck period that has not been improved to date (1, 23, 24). With the continuous development of the concept of precision medicine, it is particularly important to develop personalized treatment plans for cancer patients by grading management, and it is expected to improve the prognosis of cancer patients (25). More and more evidence shows that genetic changes and epigenetic modifications play an important role in the occurrence and progression of tumors. The use of genetic testing to assess the prognosis of patients especially the response to drug therapy has begun to be applied in the clinic. However, most of these tests rely on patient tissue and are expensive. Fortunately, recent studies have shown that many preoperative hematological markers can predict the prognosis of cancer patients (26–28). Unlike genetic testing, these hematological markers are inexpensive and readily available. In fact, most of them originate from the routine tests that every patient needs to perform on admission. Previous studies have mostly only demonstrated the prognostic value of a single hematological marker in cancer patients (29–31). However, given the complexity of the tumor microenvironment, it is difficult for a single hematological marker to fully reflect tumor characteristics and accurately predict tumor progression. In fact, although previous studies have shown that LMR has prognostic value in osteosarcoma patients, LMR has limited predictive power in our cohort (32–34). In this study, we extensively collected hematological markers that have been shown to be of prognostic value in osteosarcoma and constructed HPSS based on these markers. Compared with single hematological parameters, HPSS has more powerful predictive ability and is expected to overcome the disadvantage of unstable predictive ability of single hematological parameters. HPSS has a better predictive ability than clinical characteristics in predicting long-term patient survival. The nomogram based on HPSS has good predictive ability. The HPSS is a valid complement to clinical characteristics, and in combination with clinical characteristics enables further differentiation of patients in the clinically low-risk group. HPSS can predict the response to immunotherapy to some extent.

Tumor-associated inflammation has already been recognized as an important hallmark of cancer (35). Studies have shown that inflammatory processes promote cancer growth and transmission, as well as the activation of oncogenic signaling pathways, and are also potential mechanisms of immune resistance in cancer patients (36). And through dynamic and extensive interactions with cancer cells, immune cells also play an important role in the tumor microenvironment (37). Although the available evidence suggests a paradoxical role for neutrophils in preventing and promoting tumor progression, it is generally believed that in solid tumors, neutrophils expand in the tumor microenvironment and systemically, often associated with poor prognosis (38). In contrast, lymphocytes in the tumor microenvironment are thought to play an important role in antitumor immunity by producing cytokines and inducing tumor cell apoptosis (39). Platelets change the tumor microenvironment by secreting vascular growth factor is also considered to promote tumor cell growth and vascular proliferation, protect tumor cells from immune cell elimination, and promote tumor cell metastasis (40). As a classical inflammatory marker in cancer patients, the prognostic value of lactate dehydrogenase has been extensively studied (41). It is now generally accepted that elevated LDH levels are associated with poor prognosis in patients. In addition, some clinical trials have also demonstrated that elevated LDH correlates with response to immunotherapy, suggesting the potential value of monitoring LDH levels (42, 43). As the cornerstone of constituting the HPSS, the coefficients of SII, PLR and LDH were 0.096, 0.521 and 0.186, respectively, similarly indicating that higher SII, PLR and LDH is associated with poor prognosis in patients, further confirming previous results.

Almost all types of cancer are accompanied by a hypercoagulable state, even without thrombosis (44). Tumor cells create a hypercoagulable microenvironment by expressing coagulants, tissue factors, or inflammatory cytokines. There is a close link between the mechanism of tumor production and the system that controls blood coagulation from the early stages of the disease. The coagulation system is an important aspect of the unique vascular microenvironment for tumor proliferation and progression. The reason why tumors express substrates to induce a systemic hypercoagulable state is the discovery of circulating microparticles derived from tumor antigens or tissue factors, which are derived from the membranes of leukocytes, platelets, endothelial cells, and tumor cells after activation or apoptosis. PT test is a monitoring index of extrinsic coagulation system, which is related to fibrinogen deficiency, primary systemic and disseminated intravascular coagulation (DIC) (45). Recent studies have also shown that elevated PT is associated with poor prognosis in tumors such as liver cancer and colon cancer (46). As an important factor in the coagulation cascade and process, FIB has been shown to be associated with the invasive process of a variety of malignant tumors (29, 47). The FIB promotes angiogenesis and tumor growth by binding to growth factors such as vascular endothelial growth factor and fibroblast growth factor-2 (48, 49). In our study, the coefficients of PT and FIB were 0.051 and 0.330, respectively, indicating that they were all associated with poor prognosis of patients.

PNI is a nutritional indicator that was originally developed to predict the risk of postoperative morbidity and mortality after gastrointestinal surgery (50). Because the original PNI is complex and difficult, and to facilitate routine use in clinical practice, Onodera et al. simplified the calculation method to make it based on serum albumin levels and peripheral blood lymphocyte counts (51). Serum albumin, a commonly used parameter of nutritional status, is inversely associated with prognosis in various cancers (52). Since PNI is a combination of lymphocytes and serum albumin, it is easy to understand the relationship between PNI and survival of cancer patients. Many studies have reported that poor tumor characteristics, such as poor differentiation, large size, and metastasis, are more likely to be observed in patients with tumors with lower PNI, suggesting that low PNI may promote tumor aggressiveness and thus worsen prognosis (53, 54). In our study, the coefficient of PNI was -0.058, which is consistent with previous conclusions.

Serum ALP levels are often positively correlated with osteoblast activity, and serum ALP is common in fractures, physiological growth and bone tumors. Studies on the prognostic value of serum ALP in osteosarcoma date back even before the era of chemotherapy (55). Now, it is generally believed that elevated serum ALP is associated with a worse prognosis in osteosarcoma patients (56). As an important part of constituting the HPSS, the coefficient of ALP was 0.940, indicating that elevated ALP is associated with poor patient prognosis. This is consistent with previous findings. We believe that the introduction of serum ALP makes HPSS more suitable for patients with bone tumors and enhances its predictive ability in patients with bone tumors.

Several recent clinical trials have shown very limited benefit from immune checkpoint inhibitor therapy in patients with advanced osteosarcoma (16, 57). Therefore, careful identification of patients who may benefit from this therapy is critical. It is generally accepted that patients with higher TPS are more likely to benefit from immunotherapy (58). The results of a recent clinical trial in patients with advanced non-small-cell lung cancer showed a positive survival benefit with immunotherapy in a population with TPS of 1% or higher (22). Unfortunately there is no uniform standard in osteosarcoma, therefore, our center referred to this result to only suggest that patients with TPS ≥ 1% try immunotherapy. Unexpectedly, our results show that TPS is not effective in predicting immunotherapy response in osteosarcoma patients. We speculate that it is mainly due to the following reasons. First, only 14 patients were included in the study and all were screened by TPS. In fact, there are more patients with osteosarcoma who are not recommended to try immunotherapy because of TPS < 1%. In addition, the TPS of patients with the highest TPS was only 8%. However, the cutoff value of TPS is generally considered to be 1% versus 50% (22). Recently, an increasing number of studies have focused on the use of hematological markers to assess the efficacy of immunotherapy in tumor patients. Studies have shown that using markers such as NLR, SII and LDH to identify patients with poor immunotherapy is a potential approach (59, 60). Therefore, we explored whether HPSS, which integrates multiple hematological markers, is equally valuable in predicting immunotherapy. It is gratifying that our results show that HPSS has such potential. However, since the study included only 14 patients and the response of immunotherapy in osteosarcoma was limited, the results of HPSS in predicting response to immunotherapy need to be interpreted with caution.

Overall, compared with a single hematological marker, HPSS has stronger predictive power. In our study, both the training and validation cohorts, HPSS showed a predictive potential superior to individual hematological markers. In studies reviewing previous single hematological parameters, we found that some hematological parameters differed in their prognostic value in different cohorts. This greatly affects the clinical application of hematological markers. We speculate that due to the poor predictive ability of a single hematological markers, it cannot comprehensively respond to the complex tumor microenvironment. Therefore, we extensively collected multiple hematological markers and constructed the HPSS to improve its predictive ability. We hope that HPSS with predictive ability can overcome the disadvantage of unstable predictive ability of a single hematological markers. Through further analysis of HPSS, we found that HPSS had a weak ability to predict early patient survival, but a strong ability to predict long-term patient survival. This is in contrast to clinical features such as tumor metastasis status and pathological fracture status. Therefore, we believe that HPSS is an effective complement to clinical features and is best suited for further identification of high-risk patients among patients at low risk for clinical features.

Finally, we have the following recommendations regarding the clinical application of HPSS. For patients with primary diagnosis of osteosarcoma, it is recommended to use the hematological parameters before chemotherapy to calculate HPSS. Because the results of these hematological parameters may be affected by chemotherapy and cannot truly reflect the patient’s tumor microenvironment. However, for patients treated with immunotherapy, based on the consideration of clinical application, we believe that the calculation of HPSS should be adjusted to the detection time close to PD-L1; that is, the latest HPSS before immunotherapy should be calculated and synergized with TPS to predict the response to immunotherapy.

It must be acknowledged that our study has certain limitations. First, a retrospective study, which may lead to selection bias. Second, HPSS is composed of six hematological parameters, each of which has its own coefficient, and its calculation is more difficult than that of a single hematological marker. In addition, the hematologic markers included in this study were based on those previously shown to have prognostic value. Therefore, some markers that also have prognostic value for osteosarcoma patients may be overlooked. However, to the best of our knowledge, this is the first study to comprehensively assess the prognostic value of hematological markers in osteosarcoma, and therefore has some value. Further studies are needed to validate our conclusions. In addition, we believe that further studies are needed to assess whether HPSS can guide the treatment of osteosarcoma patients. For example, increase the frequency of follow-up lung CT for patients with high HPSS who do not develop lung metastases, or increase the chemotherapy cycles for patients with high HPSS. At the same time, the HPSS score can be appropriately considered when screening patients for prior to the application of caritizumab.

## Conclusion

Our study confirms the prognostic value of the comprehensive hematological score HPSS in patients with osteosarcoma. HPSS is an independent prognostic factor in patients with osteosarcoma. The nomogram constructed based on HPSS has good predictive ability. The HPSS is a valid addition to clinical characteristics and is suitable for further identification of high-risk patients from low clinical risk patients. HPSS has certain implications for the response to immunotherapy.

## Data Availability Statement

The raw data supporting the conclusions of this article will be made available by the authors, without undue reservation.

## Ethics Statement

The studies involving human participants were reviewed and approved by Ethics Committee of West China Hospital of Sichuan University. Written informed consent to participate in this study was provided by the participants’ legal guardian/next of kin.

## Author Contributions

'LL, YZ and CT designed the study and LL, XH, YW, ZL, CL, JL, QC jointly collected and managed the data. LL, TG co-drafted the manuscript. LM, YL, ML reviewed and corrected the manuscript. YZ and CT oversaw the entire research process. All authors contributed to the article and approved the submitted version.

## Funding

The institution of one or more of the authors (ML, LM, YZ, CT) has received, during the study period, funding from the Science and Technology Research Program of Sichuan Province (2020YFS0036), the Chengdu Science and Technology Project (2017-CY02-00032-GX), 1·3·5 project for disciplines of excellence, West China Hospital, Sichuan University (ZYJC18036), the Fundamental Research Funds for the Central Universities (20826041E4071), Post-Docter Research Project, West China Hospital, Sichuan University (20HXBH136), and Project funded by China Postdoctoral Science Foundation (2021M702342).

## Conflict of Interest

Author CL was employed by Yinfeng Gene Technology Co Ltd.

The remaining authors declare that the research was conducted in the absence of any commercial or financial relationships that could be construed as a potential conflict of interest.

## Publisher’s Note

All claims expressed in this article are solely those of the authors and do not necessarily represent those of their affiliated organizations, or those of the publisher, the editors and the reviewers. Any product that may be evaluated in this article, or claim that may be made by its manufacturer, is not guaranteed or endorsed by the publisher.
