# Supplementary material for: Hematological Prognostic Scoring System Can Predict Overall Survival and Can Indicate Response to Immunotherapy in Patients With Osteosarcoma
Source: Front Immunol. 2022 May 6;13:879560. doi: 10.3389/fimmu.2022.879560 (PMC9120642; doi:10.3389/fimmu.2022.879560)
Supplement: Supplementary file 1 [file Table_1.docx]

| Marker | Cutoff value |
| --- | --- |
| ALP | 84 |
| PLR | 191.94 |
| NLR | 2.9 |
| LMR | 2 |
| SII | 1043.33 |
| FIB | 2.3 |
| SIRI | 1.91 |
| PT | 12.1 |
| HB | 109 |
| APTT | 30.4 |
| INR | 1.02 |
| PNI | 50.4 |
| RDW-CV | 13.7 |
| RDW-SD | 47.4 |
| LDH | 160 |
| TT | 18.1 |

Supplementary table 1: Optimal cut-off values for 16 hematological markers
